# Diagnostic, Prognostic and Therapeutic Utility of MicroRNA-21 in Ischemic Heart Disease

**DOI:** 10.3390/ijms27020954

**Published:** 2026-01-18

**Authors:** Boris Burnjaković, Marko Atanasković, Marko Baralić, Aladin Altić, Emil Nikolov, Anastasija Ilić, Aleksandar Sič, Verica Stanković Popović, Ana Bontić, Selena Gajić, Sanja Stankovic

**Affiliations:** 1 Faculty of Medicine, University of Novi Sad, 21000 Novi Sad, Serbia; boris.burnjakovic@gmail.com (B.B.); 2002stasa@gmail.com (A.I.); 2 Izola General Hospital, 6310 Izola, Slovenia; atanaskovic.marko5@gmail.com; 3 Nephrology Clinic, University Clinical Center of Serbia, 11000 Belgrade, Serbia; 4 Faculty of Medicine, University of Belgrade, 11000 Belgrade, Serbia; 5 Department of Internal Medicine, Albert Einstein College of Medicine, Jacobi Medical Center/North Central Bronx Hospital, New York, NY 10467, USA; 6 Department of Surgery, University Medical Center Ljubljana, 1000 Ljubljana, Slovenia; emilnikolov99@gmail.com; 7 Center for Medical Biochemistry, University Clinical Center of Serbia, 11000 Belgrade, Serbia; 8 Faculty of Medical Sciences, University of Kragujevac, 34000 Kragujevac, Serbia

**Keywords:** ischemic heart disease, coronary artery disease, myocardial infarction, ischemic cardiomyopathy, heart failure, biomarkers, vascular inflammation, atherosclerosis, plaque vulnerability, cardiac remodeling

## Abstract

Ischemic heart disease (IHD) remains a leading cause of global morbidity and mortality despite advances in prevention, diagnosis, and therapy. Traditional clinical risk scores and biomarkers often fail to fully capture the complex molecular processes underlying atherosclerosis, myocardial infarction, and ischemic cardiomyopathy, leaving substantial residual risk. MicroRNAs have emerged as promising regulators and biomarkers of cardiovascular disease, among which microRNA-21 (miR-21) has attracted particular attention. MiR-21 is deeply involved in key pathophysiological mechanisms of IHD, including endothelial dysfunction, vascular inflammation, vascular smooth muscle cell proliferation, plaque development and vulnerability, cardiomyocyte survival, and myocardial fibrosis. Accumulating clinical evidence suggests that circulating miR-21 holds diagnostic value across the ischemic continuum, from stable coronary artery disease to acute coronary syndromes, myocardial infarction, and ischemic heart failure. Moreover, miR-21 demonstrates prognostic relevance, correlating with plaque instability, adverse remodeling, heart failure progression, and long-term cardiovascular outcomes. Preclinical studies further indicate that miR-21 represents a double-edged therapeutic target, offering cardio protection in acute ischemic injury while contributing to fibrosis and maladaptive remodeling if dysregulated. This narrative review summarizes current evidence on the diagnostic, prognostic, and therapeutic utility of miR-21 in IHD, highlighting its clinical promise as well as key limitations and future translational challenges.

## 1. Introduction

Ischemic heart disease (IHD) is a condition characterized by reduced blood supply to the myocardium, most commonly resulting from atherosclerotic narrowing of the coronary arteries. It encompasses a broad clinical spectrum, ranging from stable angina to acute myocardial infarction (MI) and ischemic cardiomyopathy, reflecting the progressive impact of chronic coronary atherosclerosis and its complications on cardiac function [1,2]. IHD is a main contributor to global cardiovascular morbidity and mortality [3], driven by both modifiable and non-modifiable risk factors, including hypertension, diabetes, dyslipidemia, smoking, age, family history/genetic factors, diet, physical activity, and the presence of other chronic diseases, etc. [4,5,6].

From a global epidemiological perspective, IHD represents one of the major causes of death and disability worldwide. In 2019, nearly 197 million individuals were estimated to be living with IHD, accounting for approximately 9.1 million deaths and a substantial number of disability-adjusted life years (DALYs), further showing its significant public health burden [7,8]. More recently, prevalence continues to rise, with over 250 million prevalent cases globally in 2021 and nearly 9 million IHD-related deaths, although age-standardized mortality rates have declined in some regions due to advances in prevention and clinical care [9]. The global impact of IHD is further shaped by demographic transitions; incidence increases with age, age-adjusted risk remains higher in men than in women [10,11], and marked regional disparities persist, particularly in low- and middle-income countries where access to preventive and therapeutic strategies may be limited [12]. All these parameters are telling us that early detection and accurate risk stratification in IHD remain challenging despite substantial clinical progress.

MicroRNAs (miRNAs) are small non-coding RNA molecules that modulate gene expression and play integral roles in numerous physiological and pathological processes, particularly in the cardiovascular system. Among them, miR-21 has been shown to influence key mechanisms, including vascular smooth muscle cell proliferation and apoptosis, cardiomyocyte growth and survival, and the regulation of cardiac fibroblast activity [13,14].

Despite the availability of established clinical risk scores, such as Framingham, SCORE, SCORE2, PROCAM, and CUORE, risk stratification across the IHD continuum remains suboptimal, as traditional algorithms often show limited performance in identifying high-risk individuals and predicting subclinical atherosclerosis [15,16]. These models often suffer from limited external validity, geographic and demographic bias, and the omission of key determinants of vascular risk, most notably molecular biomarkers that capture endothelial dysfunction and vascular inflammation. As a result, substantial residual risk persists, and up to 50% of individuals who eventually develop coronary artery disease may not be accurately identified [16,17].

Current data, therefore, highlights the need for biologically informed stratification tools capable of capturing early vascular injury and plaque vulnerability. In this context, circulating microRNAs have emerged as promising candidates that may complement traditional markers and improve individualized risk estimation [17,18]. Among them, miR-21 stands out for its well-established involvement in vascular inflammation, endothelial dysfunction, plaque development, and plaque vulnerability, positioning it as a compelling biomarker for enhanced risk prediction across the ischemic continuum [13,19].

This review aims to summarize recent progress and emerging insights into the diverse diagnostic, prognostic, and therapeutic roles of miR-21 in IHD. Beyond its associative value, miR-21 may reflect dynamic disease activity across different stages of ischemic heart disease, capturing aspects of vascular pathology not conveyed by conventional clinical markers. However, existing data are predominantly observational and heterogeneous, limiting the strength of inferences regarding clinical implementation [17,18,19].

Accordingly, this narrative review provides a comprehensive synthesis of the diagnostic, prognostic, and therapeutic potential of miR-21 across the ischemic heart disease continuum.

## 2. Methodology

This narrative review aims to synthesize and critically evaluate the existing literature on the role of microRNA-21 (miR-21) in coronary artery disease, myocardial infarction, and heart failure. In contrast to the structured format of systematic reviews, a narrative approach was adopted to accommodate the diverse nature of the evidence, including experimental, clinical, and translational research, as well as disease pathophysiology frameworks. Although primarily narrative, this review outlines explicit eligibility criteria and a transparent literature search strategy to enhance methodological rigor and ensure clarity and reproducibility.

### 2.1. Eligibility Criteria

#### 2.1.1. Inclusion Criteria

- Observational studies (case–control, cross-sectional, cohort) and clinical studies evaluating miR-21 as a biomarker or therapeutic target in CAD, MI and HF.
- Studies evaluating miR-21 as a diagnostic, prognostic or pathophysiological biomarker in cardiovascular disease.
- Human studies only.

#### 2.1.2. Exclusion Criteria

- Reviews, meta-analyses, editorials, case reports, or purely mechanistic in vitro/in vivo studies without human clinical data.
- Studies that do not investigate miR-21 within the context of CAD, MI, and HF.
- Studies that lacked quantitative miR-21 data or clearly defined clinical outcomes.
- Non-English publications.

### 2.2. Literature Search Strategy

A selective literature search was conducted across the following PubMed databases. Key search terms included the following: “microRNA-21”, “coronary artery disease”, “myocardial infarction,” and “heart failure”.

**Figure 1:**
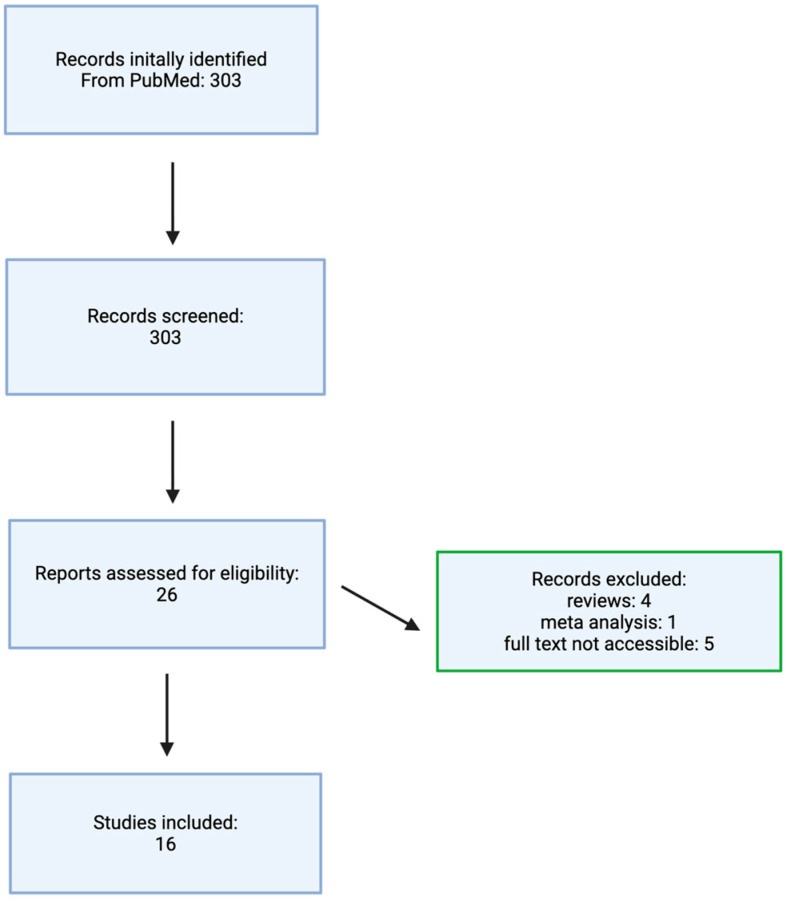
Flowchart illustrating the literature selection process. Made by authors in BioRender online application, available online: https://www.biorender.com/ (accessed on 1 December 2025).

The literature selection process is illustrated in the flowchart shown in Figure 1.

### 2.3. Search Syntax

PubMed (“microRNA-21” OR “miR-21” OR “mir21”) AND (“cardiovascular disease” OR “myocardial infarction” OR “heart failure” OR “coronary artery disease” OR “ischemic cardiomyopathy”) AND (“2010/01/01” [Date—Publication]: “2025/06/14” [Date—Publication]) AND (humans [MeSH Terms] OR humans [All Fields]).

### 2.4. Review of the Literature

The studies included in the literature review are listed in Table 1.

## 3. Pathophysiology Role of miR-21 in Ischemic Heart Disease

### 3.1. Role of miR-21 in Endothelial Dysfunction and Vascular Inflammation

Beyond its involvement in systemic inflammation, miR-21 is a crucial regulator of vascular homeostasis, acting on both endothelial cell (EC) and vascular smooth muscle cell (VSMC). In ECs, oscillatory shear stress upregulates miR-21 expression, which in turn represses peroxisome proliferator-activated receptor-α (PPARα) [36].

This downregulation relieves PPARα-mediated inhibition of activator protein 1 (AP-1), thereby promoting the expression of pro-inflammatory adhesion molecules, such as VCAM-1, and chemokines, such as MCP-1, and consequently enhancing endothelial inflammation. Furthermore, miR-21 supports endothelial function and survival by targeting phosphatase and tensin homolog (PTEN), a negative regulator of the Akt signaling pathway. PTEN suppression enhances Akt activity, leading to increased phosphorylation and activation of endothelial nitric oxide synthase (eNOS), thereby increasing nitric oxide (NO) production. This pathway promotes EC proliferation, survival, and angiogenesis, while also maintaining vascular tone [37].

The observed regulatory influence extends to macrophages within the vascular wall, where miR-21 acts as a key mediator of inflammatory balance by directly targeting and suppressing programmed cell death protein 4 (PDCD4), which is associated with increased production of anti-inflammatory cytokine IL-10 [38]. Simultaneously, miR-21-mediated PTEN suppression in macrophages steers them toward a reparative phenotype, facilitating the resolution of inflammation and tissue recovery [38,39].

Conversely, the absence of miR-21 leads to elevated PDCD4 levels and consequently amplifies the LPS-induced expression of pro-inflammatory cytokines, including TNF-α, IL-6, and IL-1β, as well as increased COX-2 levels [37,40].

Therefore, through these dual mechanisms of modulating inflammatory responses and enhancing endothelial survival and function, miR-21 emerges as a key regulator in the development of endothelial dysfunction and may significantly influence the progression of atherosclerosis [37,39].

**Figure 2:**
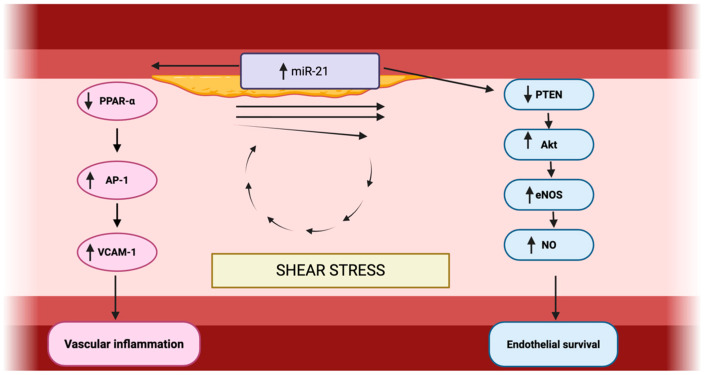
Role of miR-21 in vascular inflammation and endothelial survival. Made by authors in BioRender online application, available online: https://www.biorender.com/ (accessed on 11 January 2026).

The central mechanisms through which miR-21 modulates endothelial dysfunction, vascular inflammation, and endothelial survival are schematically summarized in Figure 2.

### 3.2. Role of miR-21 in Vascular Smooth Muscle Cell Proliferation

A central pathway through which miR-21 influences vascular disease progression is by directly stimulating vascular smooth muscle cell (VSMC) proliferation and migration, a key feature of pathological vascular remodeling. In atherosclerotic vessels, miR-21 expression is markedly elevated and localized specifically within the arterial media, overlapping with VSMCs and identifying these cells as a primary contributor to its overexpression in vascular lesions [41].

This upregulation promotes a shift in VSMCs from a quiescent, contractile phenotype to a synthetic, proliferative state, which is essential in the development of stenosis and neointimal hyperplasia. At the molecular level, miR-21 exerts its pro-mitogenic effects largely via activation of the AKT and ERK1/2 signaling cascades [42].

Additionally, miR-21 regulates cytoskeletal dynamics and cellular morphology, such as increasing cell elongation through AKT- and ERK-dependent mechanisms [41,43].

Further insight was provided by Li et al. [44], who found that reduced miR-21 expression downregulated both AP-1 (a direct target) and α-SMA in VSMCs, which was functionally linked to PDGF-induced proliferation.

Given its potent role in driving VSMC expansion, miR-21 is considered a key player in the pathogenesis of atherosclerosis and in-stent restenosis. This has inspired therapeutic strategies aimed at its inhibition; for example, stent-based local delivery of anti-miR-21 molecules has shown promise in reducing restenosis by suppressing VSMC-driven intimal thickening [43].

In summary, all of this establishes miR-21 as a critical regulator of VSMC phenotype and proliferation via the AKT/ERK axis, establishing it as both a valuable diagnostic biomarker and a compelling target for therapies designed to attenuate maladaptive vascular remodeling [41,43].

**Figure 3:**
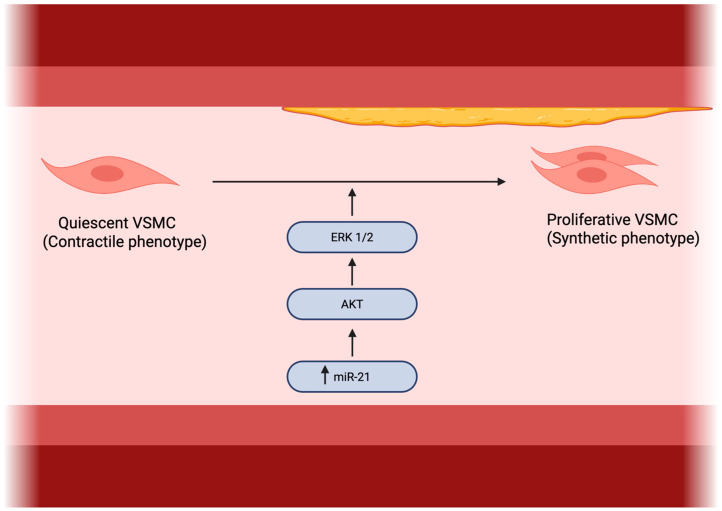
Role of miR-21 in vascular smooth muscle cell proliferation. Made by authors in BioRender online application, available online: https://www.biorender.com/ (accessed on 11 January 2026).

A schematic overview of miR-21–dependent regulation of vascular smooth muscle cell proliferation, migration, and AKT/ERK signaling is presented in Figure 3.

### 3.3. Atherosclerotic Plaque Development and Vulnerability

The role of miR-21 in atherosclerosis extends beyond vascular smooth muscle cells (VSMCs), encompassing a complex, cell-specific regulatory network that influences both plaque progression and vulnerability [39,45]. In macrophages, miR-21 exerts predominantly atheroprotective effects by modulating cholesterol homeostasis and inflammatory signaling. Loss of miR-21 disrupts this balance through derepression of mitogen-activated protein kinase kinase 3 (MKK3), which promotes post-transcriptional degradation of the cholesterol transporter ABCG1, thereby impairing cholesterol efflux and accelerating foam cell formation and lipid accumulation within atherosclerotic lesions [45,46]. This pro-atherogenic phenotype is further amplified by enhanced nuclear translocation of NF-κB in miR-21–deficient macrophages, leading to increased oxidized LDL uptake and sustained inflammatory activation [45].

Experimental studies have demonstrated that hematopoietic loss of miR-21 accelerates atherosclerosis and promotes macrophage apoptosis, resulting in larger necrotic cores and thinning of the fibrous cap, both hallmarks of plaque vulnerability [37]. Consistently, Apoe−/−miR-21−/− mice develop more advanced atherosclerotic lesions with increased macrophage infiltration and foam cell burden and exhibit a significantly higher incidence of atherothrombotic events in inducible plaque rupture models. This heightened vulnerability reflects failure to maintain fibrous cap integrity, a process critically dependent on VSMC survival and content. At the molecular level, miR-21 promotes VSMC proliferation and limits apoptosis through a feedback loop involving RE1-silencing transcription factor (REST), which is upregulated in unstable plaques and acts as an upstream repressor of miR-21. REST itself is a direct miR-21 target, establishing a regulatory circuit whereby inflammatory stimuli such as TNF-α suppress miR-21, impair VSMC survival signaling, and weaken cap stability [45].

In contrast to its protective role during chronic plaque evolution, miR-21 may contribute to acute plaque destabilization through macrophage-driven matrix remodeling. In rupture-prone human coronary plaques, macrophages exhibit elevated miR-21 expression that suppresses the endogenous matrix metalloproteinase (MMP) inhibitor RECK, resulting in increased MMP-9 activity and enhanced extracellular matrix degradation. Clinically, unstable non-calcified plaques are characterized by increased intraplaque miR-21 expression alongside reduced circulating miR-21 levels and elevated plasma MMP-9, suggesting lesion-specific sequestration and localized proteolytic activity [47].

This indicates that miR-21 regulates plaque biology in a context- and stage-dependent manner. During chronic plaque progression, miR-21 deficiency promotes macrophage apoptosis, necrotic core expansion, and reduced VSMC survival, compromising fibrous cap integrity. Conversely, under acute inflammatory or ischemic conditions, miR-21 upregulation within plaque macrophages enhances MMP activity and accelerates active plaque destabilization. This duality underscores miR-21 as a dynamic regulator rather than a uniformly protective or harmful factor in atherosclerosis [37,45].

**Figure 4:**
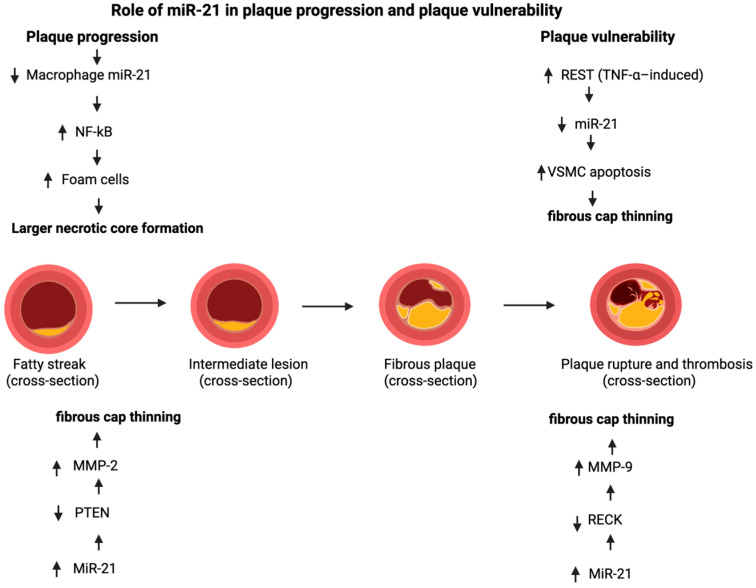
Role of miR-21 in plaque progression and plaque vulnerability. Made by authors in BioRender online application, available online: https://www.biorender.com/ (accessed on 9 December 2025).

Thus, miR-21 represents a critical regulatory node in atherosclerosis with clear cell-specific effects. While its activity in macrophages, endothelium, and the fibrous cap supports inflammation resolution, cholesterol efflux, and plaque stabilization, its pro-proliferative actions in VSMCs may facilitate lesion growth. This therapeutic paradox highlights the necessity for cell-selective and context-dependent miR-21-targeted strategies to achieve vascular protection without promoting adverse remodeling. All of the cell-specific and stage-dependent effects of miR-21 on plaque progression and vulnerability are schematically summarized in Figure 4.

## 4. Diagnostic Potential of miR-21 in Ischemic Heart Disease

### 4.1. Diagnostic Role in CAD

Accumulating clinical evidence suggests that circulating miR-21 may serve as a useful diagnostic biomarker in coronary artery disease [20,21,22].

Marketou et al. [20] demonstrated that pericoronary adipose tissue surrounding stenotic coronary segments shows markedly higher miR-21 levels (*p* = 0.012) when compared with plaque-free areas, indicating that local miR-21 expression parallels the anatomical presence of advanced atherosclerosis and may contribute to focal plaque susceptibility. Consistently, Kumar et al. [21] reported significantly elevated circulating miR-21 in CAD patients, with the highest expression observed in acute coronary syndrome; importantly, serum miR-21 achieved an AUC of 0.79 with balanced sensitivity and specificity, supporting its potential value for distinguishing CAD, particularly ACS presentations.

Conversely, Kahya Eren et al. [22] found substantially lower circulating miR-21 concentrations in young, early-onset CAD patients compared with older CAD cases, suggesting that miRNA expression patterns differ by age. These findings underscore the importance of considering age as a critical confounding variable when interpreting miR-21 for diagnostic or prognostic use in CAD.

As CAD progresses toward plaque instability, miR-21 expression patterns intensify, offering even greater diagnostic relevance in acute coronary events.

### 4.2. Diagnostic Role in ACS

Building on its diagnostic relevance in chronic coronary disease, circulating miR-21 appears to rise even further during acute plaque rupture and ischemic inflammation, indicating that its diagnostic performance may provide even greater discriminatory value in acute coronary syndromes, where markedly higher expression levels have consistently been reported [21,25].

This pronounced upregulation in ACS likely reflects the distinct pathophysiological state of acute plaque rupture and intense inflammatory activation, positioning miR-21 as a particularly promising biomarker for acute ischemic events [23,48]. Supporting this, ACS patients were directly compared to those with stable CAD, and a significant elevation in serum miR-21 in the ACS cohort (*p* < 0.001) was found, with the biomarker demonstrating an AUC of 0.769 for discriminating between these conditions. The study further linked this elevation to the mechanism of plaque instability, showing a positive correlation with MMP-9, a key enzyme in fibrous cap degradation [23]. Extending these biochemical observations, imaging-based investigations further corroborated the association between elevated miR-21 levels and plaque vulnerability.

He et al. [48] reported excellent diagnostic performance of circulating miR-21 for ACS (AUC = 1.000, *p* < 0.001), with higher levels correlating with imaging features of plaque vulnerability, including larger lipid cores, increased macrophage infiltration, and thinner fibrous caps.

Transitioning from its role in identifying acute coronary syndromes, miR-21 has also been extensively investigated as a diagnostic biomarker for myocardial infarction (MI) itself, with studies reporting its dynamic release into circulation following cardiomyocyte injury [26,27,28,29,30,31].

Research on AMI patients, often encompassing both STEMI and NSTEMI, generally indicates a significant alteration in circulating miR-21 levels following infarction.

For acute diagnosis, the studies by Zhang et al. [27] and Wang et al. [29] provide foundational evidence of miR-21′s rapid release into the circulation following cardiomyocyte necrosis. Zhang et al. [27] demonstrated a significant elevation of plasma miR-21 in AMI patients, which not only showed high diagnostic accuracy (AUC = 0.892) but also correlated strongly with established markers of myocardial necrosis: creatine kinase (CK), CK-MB, and cardiac troponin I (cTnI) [27]. This parallel rise suggests miR-21 is a core component of the early biomolecular response to ischemic injury. Wang et al. [29] reinforced this by identifying miR-21-5p as one of the most significantly upregulated miRNAs in AMI patients, noting that its level also correlated with cTnI [27,29].

In contrast to the consistent upregulation reported by Zhang et al. and Wang et al. [27,29], Xu et al. [26] observed significantly lower circulating miR-21-5p levels in AMI patients compared with unstable angina and healthy controls. Although this study demonstrated moderate diagnostic performance (AUC = 0.660), several methodological limitations likely explain this discrepancy between studies. In particular, the relatively small sample size (40 AMI patients) and the inability to precisely determine the interval between symptom onset and sampling introduce considerable variability [26].

Importantly, circulating miR-21 levels differ substantially depending on whether serum or platelet-poor plasma is analyzed, since platelet activation artificially increases serum miR-21 concentrations, whereas plasma-based sampling yields lower levels and indicating that platelet release during clotting can artificially elevate measurements and affect diagnostic interpretation [49].

Ji-Gang He et al. [50] conducted a meta-analysis of 11 case–control studies, which showed an overall pooled AUC of 0.779, indicating good potential for miR-21 to differentiate between patients with and without ACS; however, high heterogeneity among the studies was observed. Moreover, the included studies had relatively small sample sizes, which may have led to overestimation of effect sizes. Also, separate analyses of NSTEMI and STEMI were not feasible, and potential confounding factors, such as age, ethnicity, and methodological differences, could not be adequately controlled. The lack of clearly defined sampling time points further limited the assessment of temporal effects on miR-21 expression [50].

Therefore, further large-scale, well-designed studies with standardized sampling protocols, uniform miR-21 measurement techniques, clearly defined control groups, and precise timing of sample collection are required to reliably determine the diagnostic potential of miR-21 in acute coronary syndrome.

### 4.3. Diagnostic Role in Ischemic Cardiomyopathy and Heart Failure

Beyond acute myocardial injury, circulating miR-21 is increasingly recognized as a biomarker for ischemic cardiomyopathy, where its elevated levels mirror ongoing chronic ischemia-driven ventricular remodeling and continued inflammatory activation [27,29]. Multiple studies support this association.

In a recent case–control analysis, Wang et al. [51] reported significantly higher circulating miR-21 levels in ICM patients compared to healthy controls (*p* < 0.001), underscoring its diagnostic potential.

Earlier research aligns with this finding. Xie et al. [35] demonstrated that miR-21 possesses good diagnostic value for ICM, with an AUC of 0.877, 87.0% sensitivity, and 76.5% specificity. Similarly, Zhang et al. [33] found even higher discriminatory power in ischemic heart failure, reporting AUCs of 0.948 (peripheral vein) and 0.940 (coronary sinus), with sensitivity and specificity reaching 100% and 97.5%, respectively.

Collectively, these studies suggest that miR-21 exhibits diagnostic potential in ischemic cardiomyopathy and heart failure through its mechanistic links to ischemia-driven ventricular injury and remodeling, although its specificity relative to non-ischemic cardiomyopathies remains limited and requires further research.

## 5. Prognostic Role of miR-21 in Ischemic Heart Disease

### 5.1. miR-21 as a Marker of Disease Severity and Plaque Vulnerability

In addition to its diagnostic potential, circulating miR-21 demonstrates significant prognostic relevance in chronic coronary artery disease, reflecting the inflammatory and remodeling processes that underlie clinical progression. A clear clinical link between miR-21 levels and disease severity was established by Kumar et al. [21], who observed a stepwise increase in circulating miR-21 across a spectrum of angiographically confirmed CAD. Levels were highest in acute coronary syndrome, intermediate in stable angina, and lowest in non-critical atherosclerosis. This gradient suggests that miR-21 reflects not only disease presence but also the biological activity of the atherosclerotic process and overall plaque burden, offering a means to identify patients at heightened risk of progression [21].

This clinical observation is supported by experimental studies showing that loss of miR-21 accelerates hallmark features of plaque vulnerability, including enlargement of the necrotic core, macrophage apoptosis, and increased susceptibility to rupture [45,46].

Within the specific context of ACS, this prognostic relevance is further sharpened by its association with mechanisms of plaque destabilization. Elevated miR-21 has been linked to the increased activity of matrix metalloproteinases (MMP-2 and MMP-9), enzymes critical for degrading the protective fibrous cap of plaques [23,47]. This mechanistic link suggests that higher miR-21 levels may identify ACS patients harboring a more unstable plaque phenotype.

Collectively, this evidence positions circulating miR-21 as a valuable molecular indicator of an active, vulnerable atherosclerotic substrate and a heightened risk of future ischemic events [21,23,48].

### 5.2. Prognostic Role in Myocardial Infarction

The prognostic utility of miR-21 extends dynamically across the timeline following myocardial infarction (MI), reflecting different pathophysiological processes from acute injury to long-term remodeling. In the early post-MI period, Xu et al. [26] found that reduced circulating miR-21 in the infarct zone predicted major adverse cardiovascular events (MACE) within three months (AUC = 0.758). This aligns with findings by Yang et al. [52], who reported that miR-21-5p was significantly lower in STEMI patients who experienced MACE compared to those who did not. The observed negative correlation between miR-21-5p and cTnI further shows its clinical relevance. Notably, since miRNAs like miR-21-5p may be detectable earlier than cTnI during acute ischemia, they could provide a valuable window into very early cellular stress, even before definitive myocardial necrosis occurs [26].

For long-term outcomes, Rincón et al. [30] confirmed miR-21-5p as a strong independent predictor of cardiovascular death and heart failure hospitalization in a large MI cohort (HR = 2.00 per 1 SD increase), especially when its predictive power was enhanced in combination with other miRNAs such as miR-210-3p, miR-23a-3p, and miR-221-3p.

### 5.3. Prognostic Role in Ischemic Cardiomyopathy to Heart Failure

In the later stages of the ischemic continuum, miR-21 also demonstrates important prognostic relevance in heart failure secondary to ischemic cardiomyopathy. Zhang et al. [33] showed that elevated circulating miR-21 levels, measured in both peripheral venous (PV) and coronary sinus (CS) samples, were strongly associated with adverse clinical outcomes. Higher miR-21 levels independently predicted mortality (PV: RR = 1.936, *p* = 0.001; CS: RR = 1.125, *p* = 0.001) and were also linked to increased risk of rehospitalization for worsening heart failure (OR = 1.160, *p* = 0.021) [33].

These findings are consistent with observations by Wang et al. [50] who demonstrated a strong positive correlation between circulating miR-21 levels and worsening functional status, as reflected by higher NYHA class (R = 0.981, *p* < 0.0001). Similarly, Xie et al. [35] reported significant associations between plasma miR-21 levels and established markers of heart-failure severity, including N-terminal pro-B-type natriuretic peptide (NT-proBNP) and left ventricular end-diastolic volume (*p* < 0.05), further supporting its role as an indicator of advanced ventricular dysfunction [35,51].

Mechanistically, sustained elevation of miR-21 aligns with its established involvement in cardiac fibroblast activation, TGF-β-mediated extracellular matrix deposition, and maladaptive myocardial fibrosis, central processes driving adverse ventricular remodeling in ischemic heart failure [52,53].

Overall, sustained miR-21 upregulation appears to capture key fibrotic and inflammatory processes underlying adverse remodeling, reinforcing its value as a prognostic indicator in ischemic cardiomyopathy–associated heart failure [33,35,51].

## 6. Therapeutic Role of miR-21 in Ischemic Heart Disease

### 6.1. Therapeutic Role in CAD and Myocardial Infarction/Acute Coronary Syndrome

MicroRNA-21 is increasingly recognized as a bidirectional therapeutic target in CAD and MI/ACS, with its net effect critically dependent on cell type, disease stage and direction of modulation [54]. However, most studies to date remain at the mechanistic, experimental, and preclinical levels.

MiR-21 exerts cardioprotective effects in myocardial infarction models, reducing infarct size and preserving cardiac function through synergistic pro-angiogenic, anti-apoptotic, and anti-inflammatory mechanisms, mediated by multiple molecular targets including PTEN, PDCD4, NOS3, KBTBD7, STRN, and Spry-1 [55]. However, the same review emphasized a fundamental duality of miR-21 signaling, as sustained or dysregulated miR-21 activity was consistently associated with enhanced myocardial fibrosis and adverse post-ischemic remodeling, potentially offsetting its early protective effects [55].

MiR-21 is also downregulated in infarcted myocardium but upregulated in the peri-infarct border zone, and adenoviral miR-21 overexpression reduced infarct size within 24 h, at least partly through repression of PDCD4 and downstream AP-1 signaling [13]. In ischemia/reperfusion and hypoxia/reoxygenation models that forced miR-21 expression suppressed PTEN protein levels without altering PTEN mRNA, activated Akt signaling, increased the Bcl-2/Bax ratio, and reduced caspase-3–mediated apoptosis, supporting miR-21 gain-of-function as a potential strategy to limit acute ischemic cardiomyocyte death [56].

In contrast, within a CAD- and atherosclerosis-relevant immune-cell context, miR-21 was further identified as a therapeutic inhibition target, showing that predicted miR-21 targets involved in autophagy and Wnt signaling—ATG5 and LRP6—were significantly downregulated at the protein level in CAD patients, while anti-miR-21 treatment in THP-1 macrophages restored ATG5 and LRP6 expression, implicating miR-21 in macrophage-driven CAD pathobiology [56]. Extending these observations, He and Guan [47] comprehensively reviewed therapeutic targeting strategies and highlighted that miR-21 inhibition using antagomirs or locked nucleic acid–based inhibitors consistently reduced cardiac fibrosis, inflammation, and maladaptive remodeling across multiple preclinical models, while also underscoring substantial translational barriers, including non-specific tissue expression, off-target effects, delivery inefficiency, and potential systemic toxicity.

In the context of in-stent restenosis within coronary artery disease, systemic inhibition of miR-21 using locked nucleic acid anti–miR-21 was shown to reduce neointimal hyperplasia; however, this approach was accompanied by significant off-target effects in internal organs, particularly the kidneys, as evidenced by elevations in serum creatinine [57,58]. In contrast, local delivery via anti–miR-21–coated stents effectively suppressed restenosis compared to bare-metal stents, without affecting reendothelialization or causing systemic toxicity, highlighting the importance of localized, cell-selective miR-21 modulation strategies [57,58].

Limitations across these studies include their predominantly preclinical nature, small human cohorts, reliance on surrogate molecular endpoints, limited cell-specific resolution, and unresolved challenges related to safe and targeted delivery of miR-21-based therapies [47,55,56,57].

In conclusion, miR-21 represents a promising but double-edged therapeutic target in CAD and MI/ACS, offering cardioprotection during early ischemic injury while posing a risk of fibrosis and adverse remodeling if inappropriately modulated. Future research should prioritize disease-stage–stratified and cell-selective miR-21 modulation strategies, coupled with advanced targeted delivery systems, and validate these approaches in large-animal models and carefully designed early-phase clinical trials, with the ultimate goal of miR-21-targeted genetic therapy.

### 6.2. Therapeutic Role in Ischemic Cardiomyopathy and Ischemic Heart Failure

Despite major improvements in survival after myocardial infarction, post-ischemic heart failure remains highly prevalent and a leading cause of hospitalization and mortality, underscoring the need to better understand the molecular drivers of maladaptive cardiac remodeling [59].

Notably, microRNA-21 is consistently implicated in maladaptive cardiac remodeling, although evidence in patients remains indirect and derived mainly from experimental models. It is selectively upregulated in cardiac fibroblasts under stress, enhancing ERK–MAP kinase signaling, promoting fibroblast survival, and contributing to interstitial fibrosis. Inhibition of miR-21 in a mouse pressure-overload model attenuated MAPK activation, reduced fibrotic remodeling, and improved cardiac function, supporting its causal role in pathological remodeling [60]. Importantly, these findings extend to large-animal models, as intracoronary antimiR-21 delivery in a porcine ischemia/reperfusion model reduced myocardial fibrosis and hypertrophy, improved cardiac function, and suppressed inflammatory and MAPK signaling [61].

However, a key limitation of these studies is their preclinical nature, emphasizing that the clinical relevance and translational potential of microRNA-21–directed therapies remain to be determined.

## 7. Relationship of miR-21 with Other IncRNAs in Ischemic Heart Disease

Long non-coding RNAs and microRNAs regulate gene expression across multiple levels and are increasingly recognized as central mediators of cardiovascular pathophysiology, including ischemic heart disease [62]. Within this regulatory landscape, accumulating evidence suggests that miR-21 functions as part of coordinated lncRNA networks, with lncRNA GAS5 representing a clinically relevant example. In patients with coronary heart disease, circulating lnc-GAS5 is significantly upregulated, whereas miR-21 is downregulated compared with controls (both *p* < 0.001), with a strong inverse correlation observed exclusively in affected individuals (*p* < 0.001). This reciprocal expression pattern is closely linked to disease severity and inflammation, as lnc-GAS5 positively correlates with C-reactive protein and Gensini score (*p* < 0.001), while miR-21 shows negative associations with multiple inflammatory cytokines (TNF-α, IL-1β, IL-6, IL-17; all *p* < 0.05) and stenosis burden (*p* = 0.003) [63].

Beyond GAS5, circular RNAs have also emerged as relevant components of non-coding RNA networks in ischemic heart disease, owing to their stability and disease-specific expression patterns. For example, circulating circHIPK3 has been associated with myocardial fibrosis and exhibits an inverse relationship with miR-21, suggesting that combined circRNA–miRNA signatures may better reflect fibrotic remodeling than individual markers alone. Similarly, coordinated expression of lncRNAs such as MIAT together with miR-21 has been reported in ischemic injury models, supporting the concept that integration of lncRNAs and circRNAs into miR-21 centered networks may enhance the specificity of non-coding RNA profiles for disease stratification [64].

Collectively, these observations suggest that miR-21 may be embedded within broader lncRNA- and circRNA-mediated regulatory networks in ischemic heart disease. However, further mechanistic and well-powered clinical studies are required to substantiate this concept [63].

## 8. Conclusions

This narrative literature review provides an explanatory overview of the evolving literature on the diagnostic, prognostic, and therapeutic potential of microRNA-21 in ischemic heart disease. Although not systematic and without statistical analyses, this review offers a comprehensive summary of studies across all components of ischemic heart disease, including coronary artery disease, myocardial infarction/acute coronary syndrome, and ischemic cardiomyopathy progressing to heart failure.

The scarcity of studies with adequate sample sizes and the heterogeneity of methodologies limit the strength of evidence and the generalizability of findings from existing observational studies, systematic reviews, and meta-analyses in this field. Nevertheless, current research highlights the promising potential of microRNA-21, particularly as a diagnostic and prognostic biomarker, and possibly as a therapeutic target in at least one of these conditions. Its most immediate application is likely to emerge in the near future as a component of multi-biomarker panels for improved risk stratification, particularly following acute coronary syndromes. As a therapeutic target, two context-dependent strategies are being explored: the use of miR-21 inhibitors (antagomirs) to attenuate maladaptive fibrotic remodeling in heart failure, and the application of miR-21 mimics to promote angiogenesis and cardioprotection in chronic ischemic conditions. In the setting of acute myocardial infarction and ACS, this duality suggests a potential therapeutic window, with transient miR-21 upregulation aimed at limiting early cardiomyocyte death and reperfusion injury, followed by targeted inhibition during the post-infarction phase to prevent adverse fibrotic remodeling.

However, achieving clinical translation will require further mechanistic and translational research, alongside larger, methodologically homogeneous observational studies and well-designed clinical trials to ultimately establish the clinical utility of miR-21.

## Figures and Tables

**Table 1:** Overview of Included Studies Assessing microRNA-21 in Ischemic Heart Disease (*n* = 16).

Author (Year)	Study Design	Population	Disease	miR-21 Expression	Pathophysiological Mechanism	Key Findings	Conclusions	Study Limitations
Marketou et al. (2023) [20]	Case–control	49 patients with 3-vessel CAD vs. 19 valve disease controls	CAD	↑ in PCAT near lesions	↑ miR-21 in PCAT → ↑ VSMC proliferation → ↑ neointimal formation	miR-21 upregulated in PCAT at lesions (*p* = 0.012); correlated with BMI (R = 0.416)	PCAT-derived miR-21 may contribute to local plaque susceptibility	Small sample; non-healthy controls; possible subclinical atherosclerosis
Kumar et al. (2020) [21]	Case–control	78 CAD, 15 NCA, 54 healthy	CAD	↑ (~2-fold), highest in ACS	↑ inflammation, ECM remodeling, angiogenesis	AUC 0.79; sensitivity 69.4%; specificity 72.2%	Circulating miR-21 reflects CAD presence and severity	Single center; small NCA group
Kahya Eren et al. (2022) [22]	Cross-sectional	30 early-onset CAD, 30 late-onset CAD, 31 controls	CAD	↓ in early-onset CAD	↓ anti-inflammatory control → ↑ inflammatory signaling	Lower miR-21 in young CAD patients	miRNA profiles differ by age; age is a major confounder	Small sample; medication bias; limited generalizability
Darabi et al. (2016) [23]	Cross-sectional	50 ACS, 50 stable CAD	ACS	↑ in ACS	↓ RECK → ↑ MMP-9 → fibrous cap degradation	AUC 0.769; correlated with MMP-9 and hs-CRP	miR-21 reflects plaque instability in ACS	Small sample; platelet-derived MMP-9 confounding
He et al. (2019) [24]	Case–control	44 ACS, 25 controls	ACS	↑	↓ PTEN → ↑ MMP-2 → cap thinning	AUC 1.000; correlated with plaque vulnerability indices	Direct marker of plaque vulnerability	Not reported
Samadishadlou et al. (2023) [25]	Bioinformatics & ML	208 PBMC samples	MI vs. CAD	↑ miR-21-3p	↓ Treg population via TGF-β-independent pathway	ML model achieved AUC 0.96 for MI vs. CAD	miRNA signatures plus ML enable accurate classification	Not reported
Xu et al. (2023) [26]	Prospective case–control	40 AMI, 22 UAP, 22 controls	AMI	↓ miR-21-5p	↑ inflammation, impaired repair	Diagnostic AUC 0.660; prognostic AUC 0.758 for MACE	Low miR-21 predicts early AMI and short-term MACE	Small sample; onset timing unclear
Zhang et al. (2016) [27]	Cohort	17 AMI, 39 AP, 10 controls	AMI	↑ AMI > AP > controls	↑ fibroblast activation and fibrosis	AUC 0.892; correlated with CK, CK-MB, cTnI	Strong diagnostic biomarker for AMI	Small sample; no follow-up
Velle-Forbord et al. (2019) [28]	Case–control	195 initially healthy individuals	Future MI risk	↑ trend	Endothelial injury → inflammation → plaque instability	Improved Framingham AUC from 0.66 to 0.80	Enhances long-term MI prediction	Normalization issues; medication data missing
Wang et al. (2014) [29]	Cohort	26 AMI + disease controls	AMI	↑	Hypoxia-induced cardiomyocyte injury	Combined miRNAs yielded near-perfect AUC	miR-21-5p is a sensitive AMI biomarker	Small sample; limited follow-up
Rincón et al. (2022) [30]	Prospective cohort	311 MI patients	MI	↑	Regulates cardiomyocyte survival and remodeling	HR 2.00 per 1 SD; predicts HF and CV death	Strong long-term prognostic biomarker	Risk of over-adjustment
Eryılmaz et al. (2016) [31]	Prospective cohort	12 STEMI, 13 controls	STEMI	No change	Not significant	miR-423-5p and miR-30d elevated	miR-21 not useful in this cohort	Very small sample
Mi et al. (2022) [32]	Case–control	100 AMI, 50 controls	AMI	↑ (highest in total occlusion)	Correlates with ischemic injury severity	AUC 0.978 for total occlusion	Promising diagnostic and prognostic biomarker	Not reported
Zhang et al. (2017) [33]	Case–control	80 HF, 40 controls	Chronic HF	↑ (PV & CS)	↑ ERK-MAPK → fibrosis	AUC up to 0.948; predicts mortality	Strong diagnostic and prognostic value	Non-healthy controls; small sample
Wang et al. (2024) [34]	Case–control	78 ICM, 80 controls	ICM	↑	↓ apoptosis, attenuated remodeling	Strong correlation with NYHA (R = 0.981)	Closely linked to disease severity	No ROC analysis
Xie et al. (2017) [35]	Case–control	56 ICM, 60 controls	ICM	↑	↓ apoptosis, slowed remodeling	AUC 0.877	Diagnostic biomarker for ICM	Not reported

ACS—acute coronary syndrome; AMI—acute myocardial infarction; AP—angina pectoris; AUC—area under curve; CAD—coronary artery disease; CK—creatin kinase, CK-MB—creatine kinase MB isoform, cTnI—cardiac troponin I; CS—coronary sinus; CV—cardiovascular; ECM—extracellular matrix; ERK-MAPK—Extracellular signal-Regulated Kinase/Mitogen-Activated Protein Kinase; HF-heart failure; HR—hazard ratio; hs-CRP—high sensitive C-reactive protein; ICM—ischemic cardiomyopathy; MACE—major adverse coronary event; MI—myocardial infarction; miRs—microRNAs; ML—machine learning; MMP—matrix metalloproteinase; NCA—normal coronary artery; NYHA—New York Heart Association; PBMC—Peripheral Blood Mononuclear Cell; PCAT—pericoronary adipose tissue; PTEN—phosphatase and tensin homolog; PV—peripheral vein; RECK—Reversion-inducing Cysteine-rich protein with Kazal motifs; SD—standard deviation; STEMI—ST-Segment Elevation Myocardial Infarction; TGF-β—transforming growth factor beta; TREG—Regulatory T cell; UAP—unstable angina pectoris. ↑, upregulation; ↓, downregulation; → promotes/leads to.

## Data Availability

No new data were created or analyzed in this study. Data sharing is not applicable to this article.
